# Molecular characterization of an inhibitor of apoptosis protein (*IAPs*) in freshwater pearl mussel, *Hyriopsis schlegelii*

**DOI:** 10.1080/21655979.2019.1653738

**Published:** 2019-08-26

**Authors:** Di Wu, Chengyuan Wang, Wanchang Zhang, Kou Peng, Junqing Sheng, Junhua Wang, Archana Jain, Yijiang Hong

**Affiliations:** a School of Life Sciences, Nanchang University, Nanchang, China; b Jiangzhong dietary therapy technology Co. LTD, Jiangxi, China; c Key Laboratory of Basic Pharmacology and Joint International Research Laboratory of Ethnomedicine of Ministry of Education, Zunyi Medical University, Guizhou, China; d Key Lab of Aquatic Resources and Utilization of Jiangxi, Nanchang, China

**Keywords:** Apoptosis, Hyriopsis schlegelii, Hs-IAPs, IAPs, qRT-PCR

## Abstract

The inhibitor of apoptosis proteins (*IAPs*) played important roles in inhibiting the apoptosis of tumor cells by regulating caspase activity in mammals. In this study, we first cloned the full-length cDNA sequence of *IAPs* gene (designated as *Hs-IAPs*) in *Hyriopsis schlegelii*. The *Hs-IAPs* gene contained an open reading frame of 1719 nucleotides, encoding a predicted protein of 572 amino acids. qRT-PCR assay indicated that the *Hs-IAPs* gene was ubiquitously expressed in different tissues, and the highest expression level was in gills. Furthermore, we purified and obtained the recombinant protein of *Hs-IAPs* which showed a molecular weight of 82.5 kDa. We used H_2_O_2_ stimulation experiment to explore the possible function of *Hs-IAPs*. The results showed that the percentage of viable cells significantly increased following the *Hs-IAPs* concentration. These indicated that the *Hs-IAPs* may play a role in anti-oxidation causing by H_2_O_2_, and its anti-oxidative may be crucial in the process of apoptosis.

## Graphical Abstract

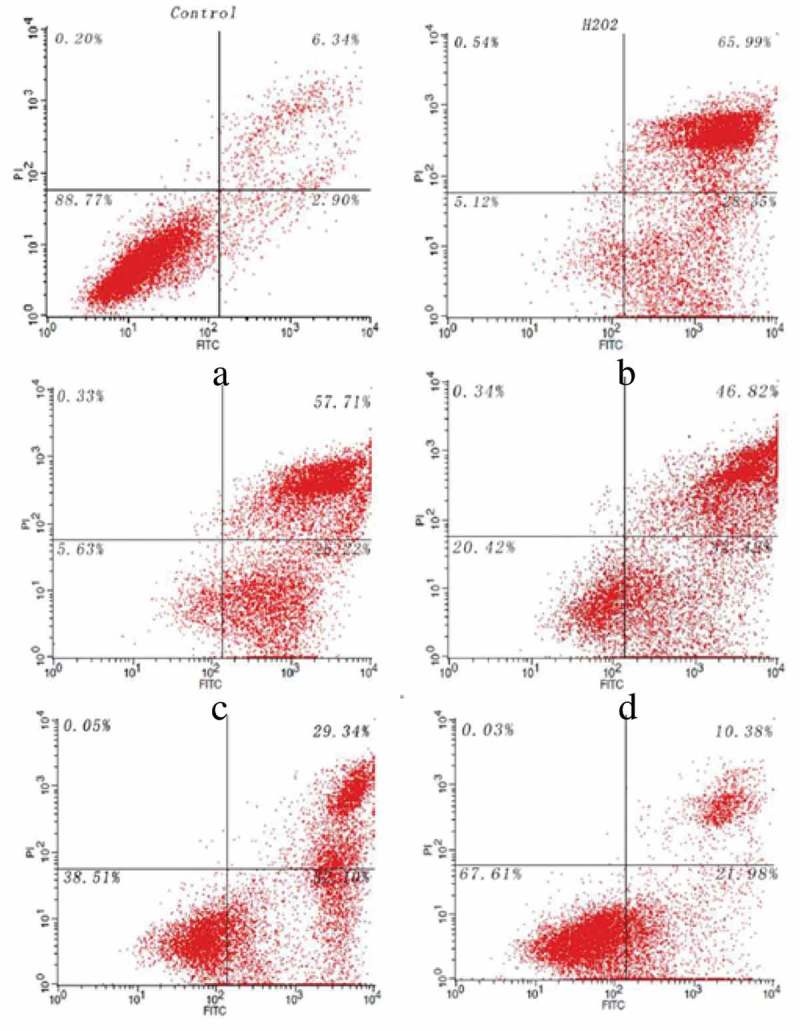

H_2_O_2_ stimulation analysis indicated that the Hs-IAPs plays a role in inhibiting apoptosis.

## Introduction

Apoptosis exists in all activities of life and life phenomena, controls embryonic development, cell proliferation and tissue regeneration [1]. The *IAPs* protein was first found in baculovirus and nuclear polyhedrosis virus [2]. From then, from lower animals including worms and insects to humans, *IAPs* have been reported. *IAPs* family is highly conserved in all reported creatures including single-cell species, like yeast, invertebrates, and vertebrates.

There are 10 family members of *IAPs*, such as *D-IAP1, D-IAP2* (Drosophila), *CeIAP* (Nematodes), *SpIAP* (Yeast) and *HIAP1, HIAP2, XIAP* and survivin (Human), etc. [3–8]. All *IAPs* family proteins have common features in suppressing cell death [9]. All of them contain one to three characteristic baculoviral *IAPs* repeats (BIR) domains containing a repeating sequence of 70 amino acids, and most of them contain a RING domain near their C-terminal, which acts as a zinc-binding motif. The BIR domain is highly conserved with Cys/His arrangement and plays important roles in inhibition of caspase activity [10,11].

Some researchers amplified four apoptosis-related factors, including *IAPs* from oysters and verified that they played important roles in the immune response [12]. It had also been found that *IAPs* existed in the different developmental stages of sea snail neurons [13]. Some papers had shown that IAPs could inhibit the expression of Caspase-3 and participate in the regulation of apoptosis [14]. So far, however, limited information about *IAPs* has been reported in freshwater mollusks.

*Hyriopsis schlegelii*, sourced from Lake Biwa of Japan, has been utilized for pearl culture in China [15,16]. In previous studies, we have found apoptotic phenomenon and known the apoptosis rate at different developmental stages in the reproductive cycle of *H. schlegeli* [17]. These results indicated that apoptosis may play a regulatory role in the gonadal development of *H. schlegelii*. These phenomena induced us to explore the possible molecular mechanism about apoptosis in *H. schlegelii.*

So, in this paper, an *IAPs* gene from *H. schlegelii* (*Hs-IAPs*) have been identified, and its tissue expression profiles have also been examined. Furthermore, we explored its possible anti-oxidation function through H_2_O_2_-induced experiment. We hypothesized that *IAPs* gene from *H. schlegelii* shared some conserved structures of *IAPs* family and had unique expression profiles in tissues (Hypothesis 1). Meanwhile, we expected *Hs-IAPs* protein to played an important role in anti-apoptotic in enriched H_2_O_2_ environment (Hypothesis 2).

## Materials and methods

### Animals/materials

Samples of *H. schlegelii* were collected from culture base in Fuzhou (Jiangxi Province, China). All of the *H. schlegelii* cultured in freshwater tanks and maintained at an ambient temperature under natural light conditions. Samples used during this study were three-year-old *H. schlegelii*.

### Molecular cloning and phylogenetic analysis

The partial sequence of *IAPs* gene was screened from gonadal transcriptome of *H. schlegelii*. The 5ʹ and 3ʹ ends were cloned by rapid amplification of cDNA ends (RACE) approach. The primers for RACE were listed in Table 1. Nucleotide and deduced amino-acid sequences were analyzed using Blast (http://www.ncbi.nlm.nih.gov/blast) in NCBI. Multiple sequence’s alignment and phylogenetic tree were performed through ClustalW Multiple Alignment program (http://www.ebi.ac.uk/clustalw). The amino acid’s identities of BIR and RING domain were calculated using FASTA programs.

**Table 1.** Primer sequence list for the preparation of *Hs-IAPs.*

Primer name	Primer sequence (5ʹ–3ʹ)	Usage
5ʹ*Hs-IAP*-1	5ʹ-AACCGAAACATCCGCATTACTCCG-3’	5´ RACE
5ʹ*Hs-IAP*-2	5ʹ-ATGCCGTATGAGAGTAGGGTCCGAG-3’	5´ RACE
3ʹ*Hs-IAP*-1	5ʹ-AGAGGTTTCGGATCCTAGCAATGACAC-3’	3´RACE
3ʹ*Hs-IAP*-2	5ʹ-CGATGCCTCTACGCAATATTCGGTCAG-3’	3´RACE
	5ʹ-GAAACGAGGAACAATCAGG-3’	RT-PCR
	5ʹ-GTCAGAACGGAGTAATGCG-3’	RT-PCR
P136F	ATgaattcATGGATAATACTACGGATCG	Vector construction
P136R	AgtcgacTCACGATACGTACGCCCTTA	Vector construction

### qRT-PCR

Total RNA was isolated from different tissues in the 3-year-old *H. schlegelii* using TRIzol (Invitrogen) reagent. RNA purity and integrity was further confirmed by 1% agarose gel electrophoresis. cDNA was synthesized using Oligo-(dT)_18_ primers with M-MLV Reverse Transcriptase (Promega).

Then, we constructed a recombinant plasmid to obtain the *IAPs* recombinant protein. The primers for plasmid construction were also listed in Table 1. The Hs-IAPs PCR product was then cloned into *EcoR* I and *Sal*Ⅰ site of pET32a. The pET32a-IAPs plasmids were transformed into BL21 (DE3) cells, and then induced with 1 mM IPTG at 25°C with speed at 220 revolutions per minute. The bacterial cells were obtained by centrifugation, resuspended in the lysis buffer, and broken by ultrasonic. At last, the recombinant proteins in the supernatant were separated using His-bind nickel column chromatography (Novagen, Germany). The recombinant protein samples were separated and compared by 12% SDS-PAGE and stained with Coomassie brilliant blue R250 (Beyotime, China).

### Western blot analysis

Purified recombinant *Hs-IAPs* proteins were performed on 12% SDS-PAGE gels by electrophoresis and then blotted to polyvinylidene difluoride (PVDF) membranes. After blocker with PBST, members were incubated with rabbit serum with antibody of *Hs-IAPs* at a dilution of 1:20000. Washed three times with PBST, membranes were further incubated with goat anti-rabbit horseradish peroxidase (HRP)-conjugated IgG at a dilution of 1:200. Then, members were checked according to HRP-DAB detection Kit instructions.

### Detection of H_2_O_2_-induced apoptotic by flow cytometry

In this experiment, Hela cells were incubated in DMEM (Gibco) at 5 × 10^5^ cells/mL. Then, the cells were incubated for 24 h. Five experimental panels were designed as follows: (A) Control: Add equal volumes of a medium. (B) H_2_O_2_: Cells were treated with 4 mmol/L H_2_O_2_ for 2 h. (C) Group 1：Cells were incubated with 0.5 mmol/L *Hs-IAPs* protein and 4 mmol/L H_2_O_2_ for 2 h. (D) Group 2：Cells were incubated with 1 mmol/L *Hs-IAPs* protein and 4 mmol/L H_2_O_2_ for 2 h. (E) Group 3: Cells were incubated with 2 mmol/L *Hs-IAPs* protein and 4 mmol/L H_2_O_2_ for 2 h. (F) Group 4：Cells were incubated with 4 mmol/L *Hs-IAPs* protein and 4 mmol/L H_2_O_2_ for 2 h.

The cells were digested with trypsin and washed for three times with PBS (phosphate buffer solution (Solarbio, China)) according to the manufacturer’s protocol provided in the Annexin V-FITC kit (BD Sciences, China) [18].

Quantitative cells (1 × 10^5^) were analyzed by flow cytometry. The annexin V-FITC fluorescence (525 nm) is measured from the FL1 channel (green) and PI fluorescence (617 nm) is measured from the FL2 channel (red). Different cell types are shown in different regions (i) live cells (Low Left) annexin V-negative and PI-negative, (ii) early apoptotic (Low Right) annexin V-positive and PI-negative, (iii) late apoptotic and necrotic (Up Right) annexin V-positive and PI-positive.

## Results

### Full-length sequence and phylogenetic analysis

**Figure 1.**
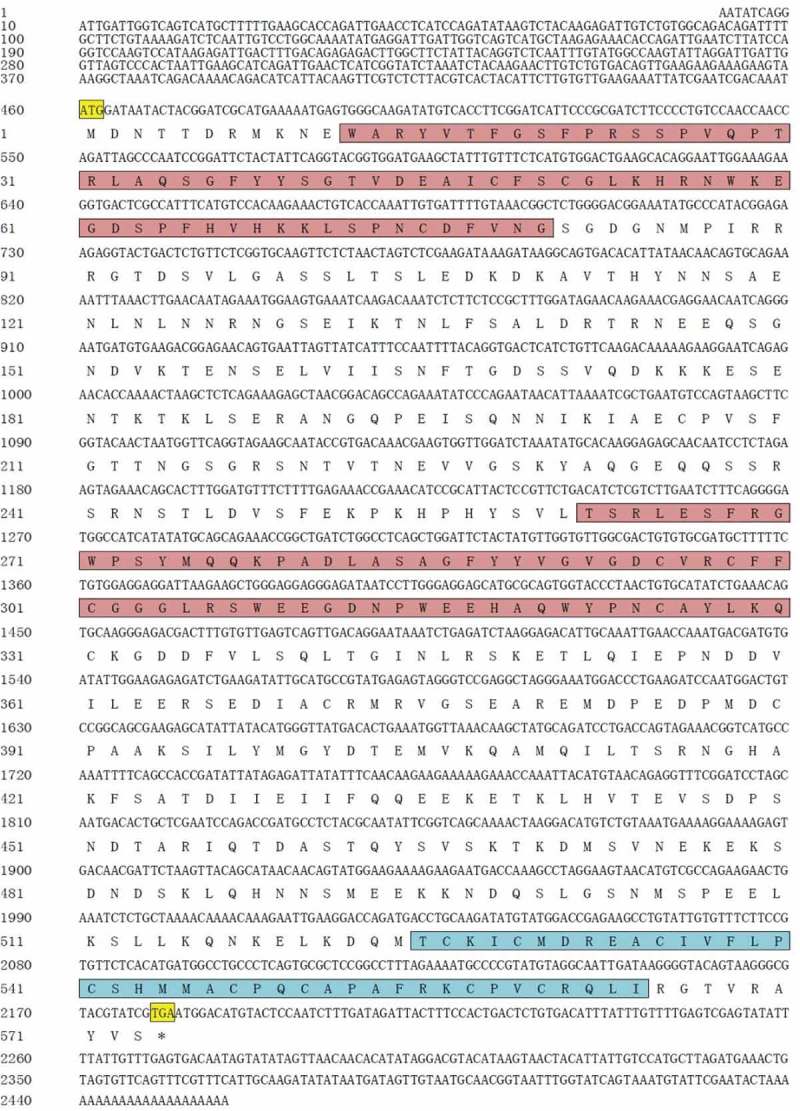
Nucleotide sequences of the five-flanking regions, cDNA and predicted amino-acid sequence of *IAPs* gene from *H.schlegelii*. Nucleotides are indicated above and numbered to the left of each lane (upper row). The deduced amino-acid sequence is shown below the nucleotide sequence. The start codon is signed by yellow grid. The stop codon is indicated by an asterisk. The BIR domains is signed by purple grid, and the RING domains is signed by blue grid.

The sequences obtained by RACE were assembled to a full-length of the *Hs-IAPs*. The complete cDNA sequence of *Hs-IAPs* contained 2,452 bp, which consisted of an ORF of 1719 bp encoding 573 aa. Two BIR domains (from 12th aa to 80th aa and from 262th aa to 330th aa) and a RING domain (from 525th aa to 564th aa) near its C-terminus were predicted (Figure 1).

Phylogenetic analysis showed that *H. schlegelii* clustered with *Crassostrea gigas* (Accession number: WKC26950.1) and *Aplysia californica* (XP_005113371.1). However, *Capitella teleta* (ELU18724.1), *Danio rerio* (XP_005162039.1), *Drosophila grimshawi* (XP_001983743.1) and *Tribulium castaneum* (AEQ93552.1) formed a separate clade.

**Figure 2.**
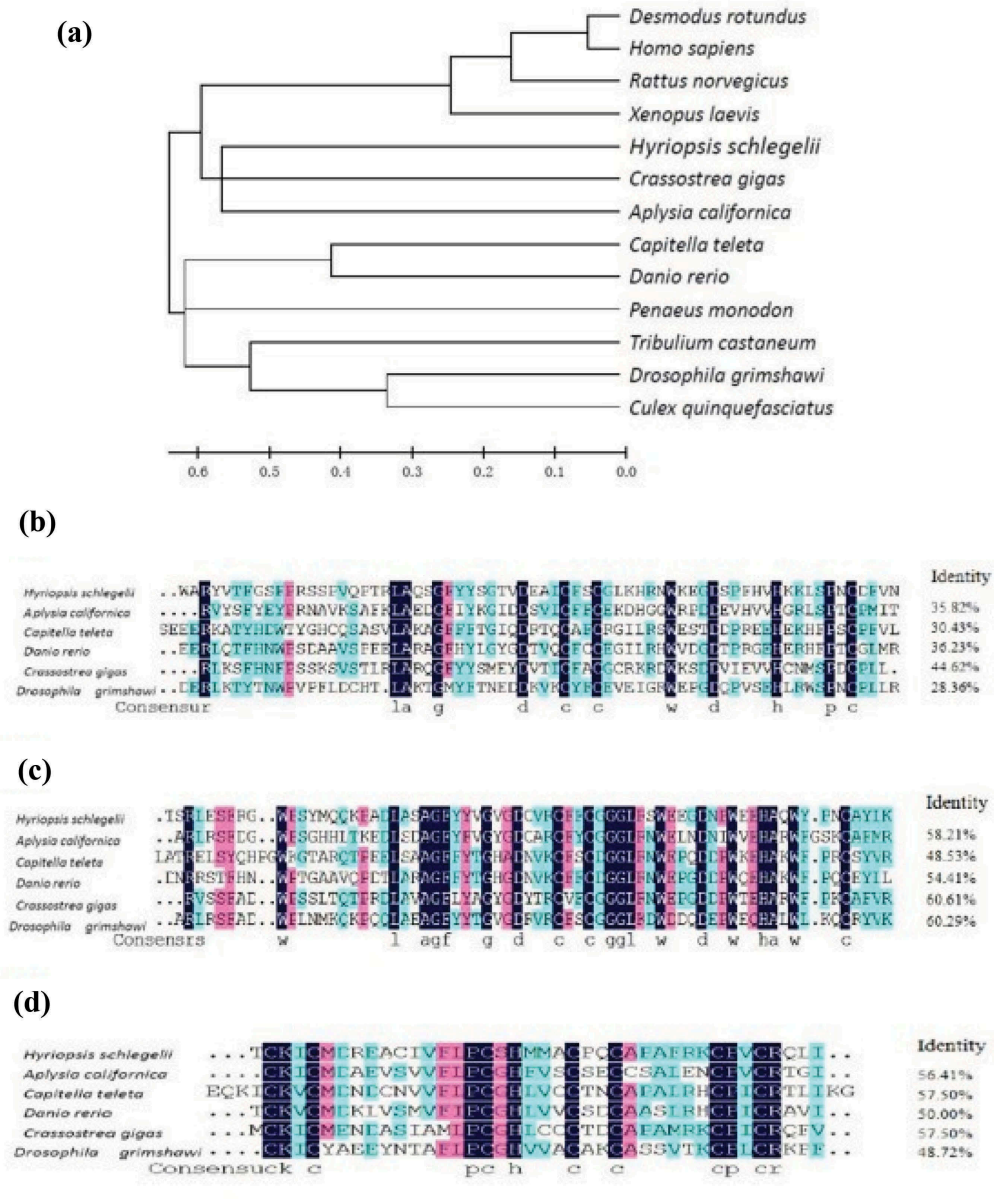
Phylogenetic tree of Hs-IAPs from different organisms based on amino-acid sequence. Distances are used to construct the phylogenetic tree and bootstrap values based on 1000 replicates. The bottom scale refers to percentage divergence (p-distance). Sequence alignments of the BIR1 (B), BIR2 (C) and RING (D) domains of *H.schlegelii* with the corresponding domains of other IAP family members are shown. The GenBank accession numbers of sequences used for the alignments are: *Desmodus rotundus* (JAA 48,496.1), *Homo sapiens* (NP 001157.1), *Rattus norvegicus* (NP 07677.3), *Xenopus laevis* (NP 001086733.1), *Penaeus monodon*（ABO38431.1）,*Tribulium castaneum*（AEQ93552.1）, *Culex quinquefasciatus* (XP_001843370.1), *Aplysia californica* (XP_005113371.1), *Capitella teleta* (ELU18724.1), *Danio rerio* (XP_005162039.1), *Crassostrea gigas* (WKC26950.1), *Drosophila grimshawi* (XP_001983743.1).

ClustalW analysis showed that the BIR domains showed the highest identity (60.61%) with *C. giga*s and the lowest identity (48.53%) with *C. teleta* (Figure 2(c)). Meanwhile, the RING domains of *Hs-IAPs* showed the highest identity (57.50%) with *C. giga*s and the lowest identity (28.36%) with *D. grimshawi* (Figure 2(b and d)).

### Tissue distribution of the Hs-IAPs

**Figure 3.**
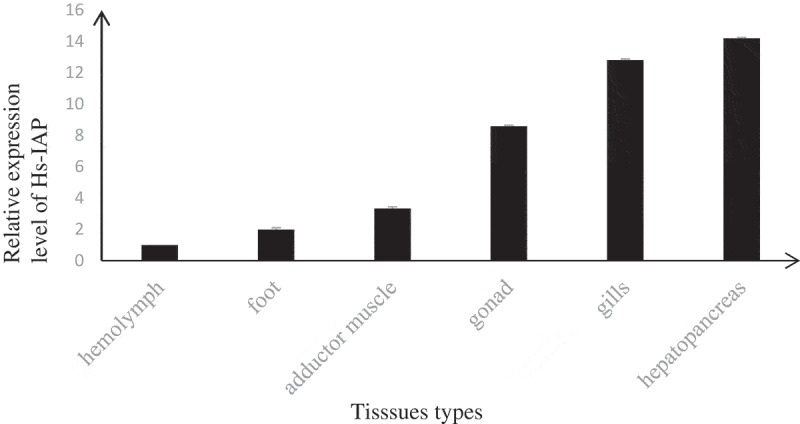
*Hs-IAPs* transcript levels in hemolymph, foot, adductor muscle, gonad, gills, and hepatopancreas were normalized to that in hemolymph using real-time PCR. In the experiments, β-actin gene was used as an internal control to calibrate the cDNA template for all the samples.

RT-PCR showed that the *Hs-IAPs* was expressed in all the examined tissues, like hemolymph, adductor muscle, gonad, foot, gills, and hepatopancreas (Figure 3). The highest expression of *Hs-IAPs* was in hepatopancreas and gills, while the lowest was in hemolymph.

### Recombinant protein induction and identification

**Figure 4.**
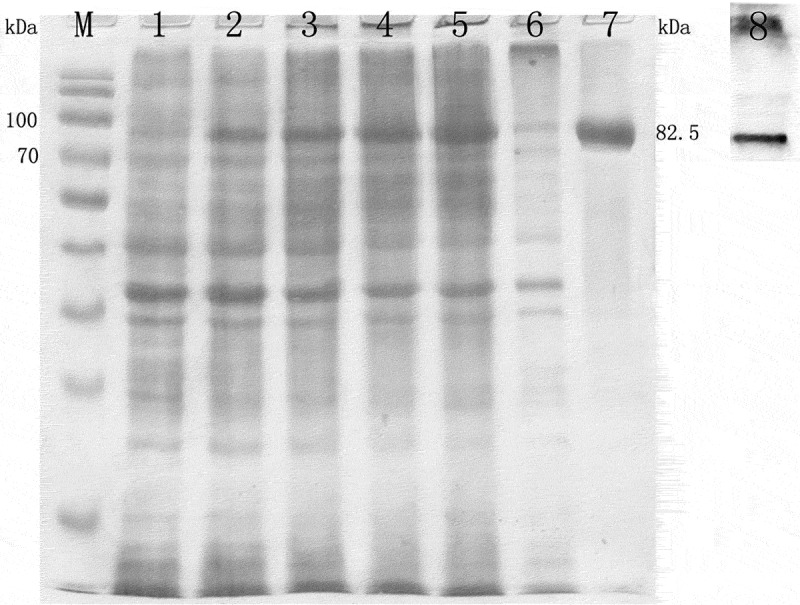
SDS-PAGE and western blot analysis of Hs-IAPs. Bacterial proteins and recombinant fusion proteins were separated on 10% SDS-PAGE gels. M, molecular weight marker; Lane 1,non-induced; Lanes 2–5, 1mM IPTG, induced expression for 1–4 h of Hs-IAPs.; respectively; Lane 6, supernatant; Lane 7, purified recombinant proteins；Lane8，Western blot analysis of Hs-IAPs.

The recombinant *Hs-IAPs* proteins were purified with Ni-NTA affinity columns from the culture supernatant. Purified recombinant *Hs-IAPs* proteins with an expected molecular mass was shown, further confirming by SDS-PAGE and Western blotting using anti-*Hs-IAPs* rabbit serum (Figure 4).

### The results of flow cytometry

**Figure 5.**
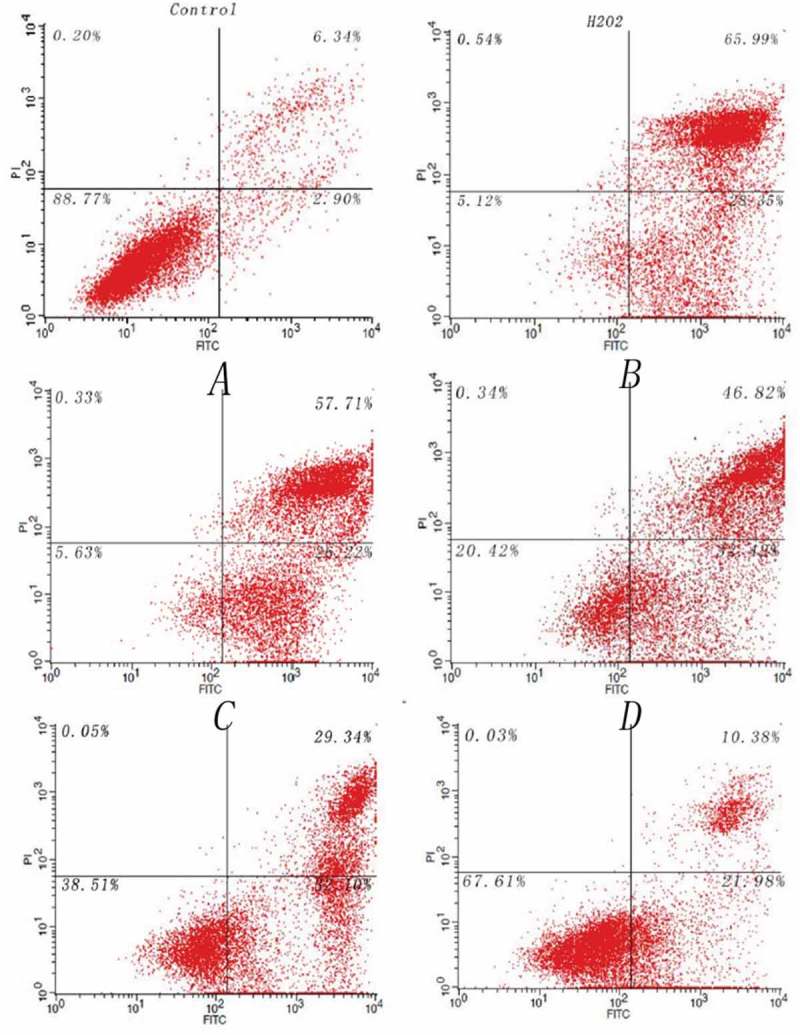
Flow cytometry analysis of Hs-IAPs-H_2_O_2_ experiments. This included six groups, control group, H_2_O_2_ stimulation group containing A, B, C, and D which were stimulated with four different concentrations of Hs-IAPs.

About 88.77% of the HeLa cells were viable cells within the control group (Figure 5). In the control group, the percentage of apoptotic and necrotic cells reached 94.34%, when stimulating the HeLa cells with H_2_O_2_ for 4 h. In other four experimental groups, the HeLa cells were exposed to both *Hs-IAPs* protein and H_2_O_2_. The percentage of viable cells was significantly increased from 5.63% to 67.61% following the *Hs-IAPs* concentration, while the percentage of apoptotic and necrotic cells decreased from 83.93% to 32.36% (Figure 5).

## Discussion

The *IAPs* could inhibit cell death pathway through its anti-apoptotic activity [3,4,19]. In this study, the *IAPs* was first cloned and characterized in freshwater pearl mussels, *H. schlegelii*. The *Hs-IAPs* contained two BIR domains and one RING domain near its C-terminus. All of *IAPs* family proteins contained one to three characteristic BIR domains, and also contained RING-finger domain. The BIR and RING-finger domains play important roles against apoptosis [20–22]. *IAPs* could inhibit apoptosis by combining and interacting between the BIR motifs and other apoptotic-related proteins [23–25]. In Pacific oyster, *IAPs* also had one RING and one or two BIRs domain [12]. The CfIAP1 protein had two BIR domains, while the CfIAP2 protein only had one BIR domain and one RING domain [14]. Meanwhile, in *Penaeus monodon, PmIAPs* contained three BIR domains [23]. All these differences of structure of *IAPs* may be related to its different functions.

In the present study, phylogenetic analysis indicated that the Hs-IAPs clustered with their homologues of the *A. californica* and *C. gigas*. The Hs-IAPs BIR2 was highly conserved by the amino-acid sequence alignments (Figure 2(c and d)). The motif ‘CX_2_CX_16_HX_6_C’ (C represented cysteine; H represented histidine; and X represented any amino acids) was specific to all the tested sequences. In some studies, four *IAPs* had been reported in oyster and verified that it played a role in immune response [26]. It had also been found that *IAPs* existed in different developmental stages of nerve cells in *A. californica* [13].

The *Hs-IAPs* mRNA was detected in all tested tissues. Interestingly, we found that the highest *Hs-IAPs* mRNA expression level was in the hepatopancreas and gills. In *C. giga*, the highest *CgIAP2* expression levels also presented in the gills [26]. The researchers thought the gills were energy exchange places between the host and its living environment. Furthermore, the stimulation experiment with *V. alginolyticus* confirmed that *CgIAP2* played an important role in defending against pathogens [26]. Thus, all these results indicated that *Hs-IAPs* might play important roles in resistance to some stressors in environment.

For exploring the possible function of *Hs-IAPs*, we conducted the experiment to verify the combination effect of *Hs-IAPs* and H_2_O_2_ on the Hela cells. The result displayed that the apoptosis and necrocytosis percentage of the Hela cells decreased following *Hs-IAPs* concentration increase. In the experimental group D, the *Hs-IAPs* concentration had the potent effect of anti-oxidant, and the percentage of apoptotic and necrotic cells was declined to 32.36%. This result indicated that *Hs-IAPs* protein could alleviate the effect of H_2_O_2_ in the environment and decreased the rate of cells apoptosis.

## Conclusions

In summary, an *IAPs* gene, designated as *Hs-IAPs*, was identified for the first time in *H. schlegelii. Hs-IAPs* had two BIR domains and one RING domain. In phylogeny, it clustered with the *A. californica* and *C. gigas*. Furthermore, the highest *Hs-IAPs* mRNA expression level was in the gills. All these results suggested that it could be a member of *IAPs* family. The Hela cells experiment indicated that *Hs-IAPs* may play a role in anti-oxidation causing by H_2_O_2_.

## Highlights

1. For the first time, this study cloned the full-length cDNA sequence of Hs-IAPs.

2. qRT-PCR assay indicated that the Hs-IAPs gene was expressed in different tissues.

3. The Hs-IAPs plays a role in inhibiting apoptosis.
